# The mutational pattern of homologous recombination repair genes in urothelial carcinoma and its correlation with immunotherapeutic response

**DOI:** 10.1002/cam4.6725

**Published:** 2023-11-20

**Authors:** Junru Chen, Yanfeng Tang, Huanhuan Liu, Guangxi Sun, Haoyang Liu, Junjie Zhao, Zilin Wang, Yanrui Zhang, Feng Lou, Shanbo Cao, Jiayue Qin, Huina Wang, Banghua Liao, Hao Zeng

**Affiliations:** ^1^ Department of Urology, Institute of Urology, West China Hospital Sichuan University Chengdu Sichuan China; ^2^ Acornmed Biotechnology Co., Ltd. Beijing China; ^3^ Acornmed Biotechnology Co., Ltd. Tianjin China

**Keywords:** homologous recombination repair, immunotherapy, tumor immune microenvironment, urothelial carcinoma

## Abstract

**Background:**

The mutational pattern of homologous recombination repair (HRR)‐associated gene alterations in Chinese urothelial carcinoma (UC) necessitates comprehensive sequencing efforts, and the clinical implications of HRR gene mutations in UC remain to be elucidated.

**Materials and Methods:**

We delineated the mutational landscape of 343 Chinese UC patients from West China Hospital and 822 patients from The Cancer Genome Atlas (TCGA) using next‐generation sequencing (NGS). Data from 182 metastatic UC patients from MSK‐IMPACT cohort were used to assess the association between HRR mutations and immunotherapy efficacy. Comprehensive transcriptomic analysis was performed to explore the impact of HRR mutations on tumor immune microenvironment.

**Results:**

Among Chinese UC patients, 34% harbored HRR gene mutations, with *BRCA2*, *ATM*, *BRCA1*, *CDK12*, and *RAD51C* being the most prevalently mutated genes. Mutational signatures contributing to UC differed between patients with and without HRR mutations. Signature 22 for exposure to aristolochic acid was only observed in Chinese UC patients. The presence of HRR mutations was correlated with higher tumor mutational burden, neoantigen burden, and PD‐L1 expression. Importantly, patients with HRR mutations exhibited significantly improved prognosis following immunotherapy compared to those without HRR mutations.

**Conclusions:**

Our findings provide valuable insights into the genomic landscape of Chinese UC patients and underscore the molecular rationale for utilizing immunotherapy in UC patients with HRR mutations.

## 1 INTRODUCTION

Urothelial carcinoma (UC), primarily originating from the urinary tract, ranks as the 10th most common malignancy worldwide.^1^ UC exhibits an extremely aggressive nature in the advanced stage, with a 5‐year survival rate of less than 5%.^2^ Platinum‐based chemotherapy has been the standard first‐line systemic treatment for patients with locally advanced and metastatic UC (mUC) for several decades.^3^, ^4^, ^5^ However, no substantial improvement in the efficacy of the chemotherapy has been observed, and approximately 50% of patients are ineligible for cisplatin treatment.^6^ Although several prospective trials have shown survival benefits of immunotherapy in patients ineligible for cisplatin or progressing after platinum‐based chemotherapy, a large proportion of them failed to respond.^7^, ^8^, ^9^ While high PD‐L1 expression could suggest a better prognosis in patients treated with immunotherapy under certain circumstances, it is not a robust predictive biomarker of response to immunotherapy in mUC.^9^, ^10^, ^11^ Thus, the identification of novel biomarkers for immunotherapy efficacy remains of great interest and is urgently needed.

Homologous recombination repair (HRR) is one of the primary mechanisms that ensure the accurate repair of DNA double‐strand breaks. HR deficiency (HRD), which leads to increased genome instability and drives tumorigenesis, is frequently observed in various malignancies, including breast, ovarian, and pancreatic cancer.^12^, ^13^, ^14^ One recent study reported the frequency of germline HRR mutations in Chinese upper tract urothelial carcinoma (UTUC) patients and revealed HRR mutations were predictive for recurrence.^15^ However, the mutational landscape of HRR genes in overall UC in Chinese populations remains incompletely understood. Whether there is a difference in the mutational pattern of HRR genes between UTUC and urothelial carcinoma of the bladder (UCB) is unknown. Several previous studies have reported an association between HRD and increased immune cell infiltrations in ovarian and breast cancer, suggesting a potential to benefit from immunotherapy.^16^, ^17^ Furthermore, it is yet to be determined whether there are any associations between HRR gene mutations and tumor immune profiles, as well as the responses to immunotherapy, in UC.

Therefore, the objectives of this study were to investigate the prevalence and mutational patterns of HRR gene mutations in Chinese UC patients. Additionally, we aimed to assess the impact of HRR gene mutations on tumor immune characteristics and treatment outcomes in UC patients who received immunotherapy.

## 2 MATERIALS AND METHODS

### 2.1 Patient enrollment and study design

A total of 343 Chinese UC patients with genomic data were recruited at West China Hospital between January 2015 and April 2020, including 118 UTUC patients and 225 UBC patients. Additionally, sequencing and clinical data from 822 patients with UC in the TCGA database and 182 mUC patients with immunotherapy from the MSK‐IMPACT cohort were obtained via the cBioPortal data portal (http://www.cbioportal.org/). The patient characteristics are summarized in Tables S1 and S2. This study was approved by the Ethical Committee of West China Hospital (Approval Number: 2020 [1009]). All participants provided informed consent.

### 2.2 HRR genes

HRR genes of interests included *ATM*, *BARD1*, *BRCA1*, *BRCA2*, *BRIP1*, *CDK12*, *CHEK1*, *CHEK2*, *FANCL*, *PALB2*, *PPP2R2A*, *RAD51C*, *RAD51B*, *RAD51D*, and *RAD54L*, based on their core functions within the HRR pathway.^18^

### 2.3 Library preparation and next‐generation sequencing

DNA was extracted from tissue and matched blood controls using the QIAamp Genomic DNA kit (Qiagen GmbH) per instructions. Sequencing libraries were established based on instructions from Illumina (Illumina, Inc.).^19^ Next‐generation sequencing (NGS) was performed using the Acornmed 808 panel as previously described.^15^ The target‐enriched libraries were performed with the Illumina HiSeq2500 NGS platform (Illumina, Inc.). In addition, the sequencing depth was no less than 10,000×. Burrows‐Wheeler Alignment tool was used to align the sequence reads. MuTect2 was used to identify SNVs and indels.^20^ The following parameters were used: (1) the number of mutant allele reads no fewer than 10, (2) coverage for normal samples of 50× and tumor samples of at least 100×, (3) a mutation allele frequency of at least 1%, (4) an allele frequency (AF) according to the database Exome Aggregation Consortium (ExAC)^21^, ^22^ no less than 0.5%, (5) all silent mutations ignored, and (6) a tumor purity of at least 20%.^21^, ^22^, ^23^ Copy number variant (CNV) was analyzed by CONTRA software.^24^

### 2.4 Calculation of tumor mutational burden (TMB)

TMB was calculated by the counts of somatic, coding, and indel mutations and base substitutions per megabase (Mb) of the genome examined. For the Chinese cohort assessed using the Acornmed 808 panel, TMB was computed by the counts of mutations counted/2 Mb. For the TCGA data, the sample TMB estimated by whole‐exome sequencing (WES) was computed by the counts of mutations counted/38 Mb, with 38 Mb chosen as it is the generally accepted length of a human exon.

### 2.5 Immunohistochemistry (IHC) analysis

Immunostaining of PD‐L1 was performed using a 1:100 dilution of a rabbit monoclonal antibody (Cat No. ab205921, Abcam). The PD‐L1 tumor proportion score (TPS) is representative as the percentages of tumor cells that are positive for membrane staining at any intensity based on the clonal 28–8 IHC test criteria.^25^, ^26^ Positive PD‐L1 was defined as TPS ≥1% in this study.

### 2.6 Mutational signature analysis

Mutational signature analysis was used to classify the SNVs for each sample, which was separated into 96 base‐substitution types based on the Bayesian nonnegative matrix factorization (NMF) using the MutationalPatterns R package.^27^, ^28^ The discovered signatures were contrasted with 30 COSMIC signatures according to “ward. D2” linkage. Signatures with similarity values less than 0.80 were defined as new signatures.^29^

### 2.7 Associations between HRR mutations and immune cell abundance and immune signature genes

To determine the impact of HRR mutations on the immune microenvironment, the abundance of 22 immune cell types was calculated by the CIBERSORTx web portal (https://cibersortx.stanford.edu/) from the TCGA dataset. The immune signature genes were selected according to previous studies.^30^, ^31^

### 2.8 Statistical analyses

SPSS 22.0 software (IBM Corporation) was used for statistical analyses. Fisher's exact test was used for comparisons of categorical variables, and false discovery rate (FDR) correction was applied. The Mann–Whitney *U* test was performed to compare continuous variables. Kaplan–Meier survival plots and log‐rank test were used for survival analysis. All *p* values were two‐sided and *p* < 0.05 was considered statistically significant.

## 3 RESULTS

### 3.1 The landscape of HRR mutations in urothelial carcinoma

**FIGURE 1:**
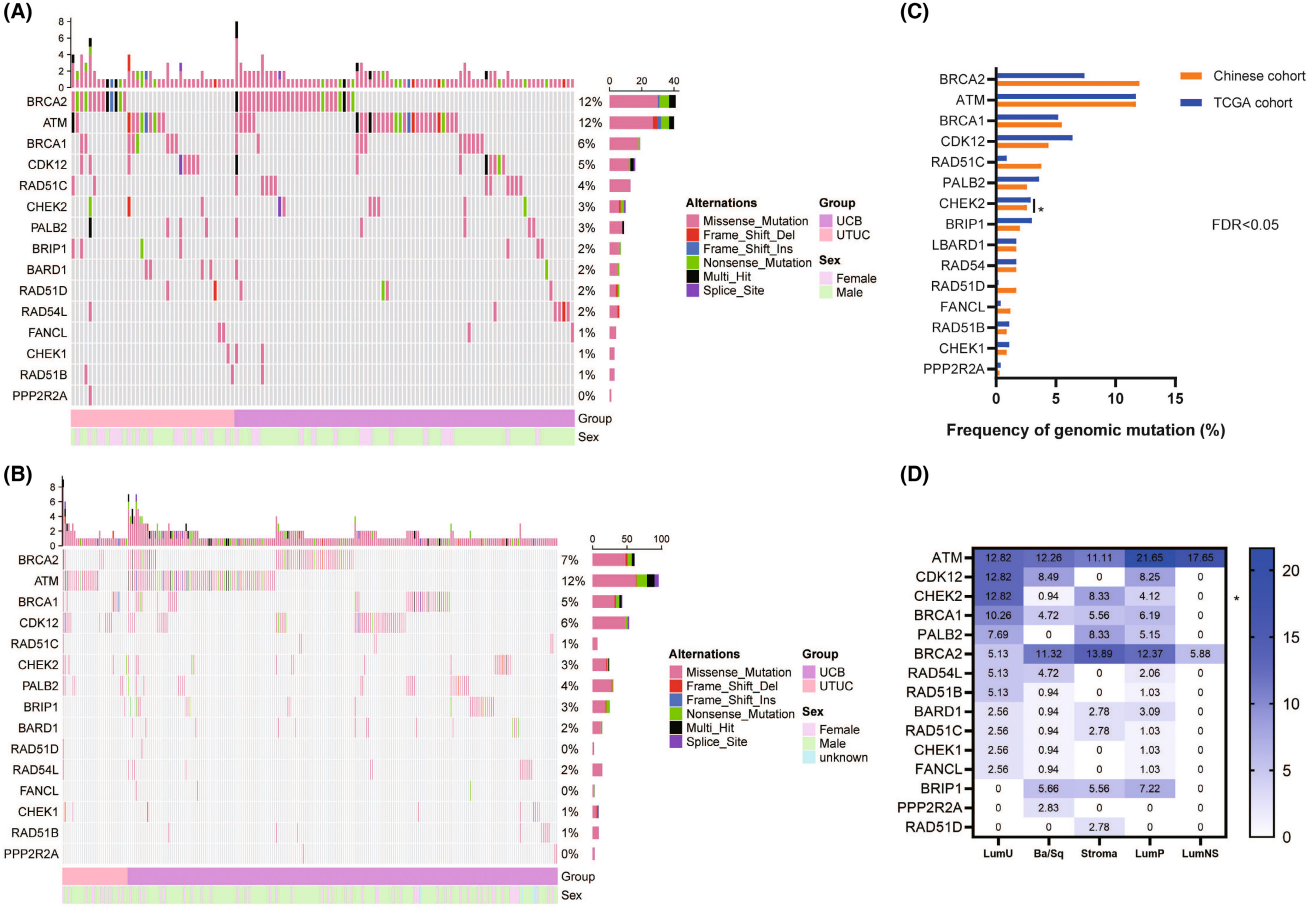
The landscape of frequently mutated HRR genes in UC. (A) The landscape of mutated HRR genes in the Chinese group. Columns and rows represent patients and genes, respectively. (B) The landscape of mutated HRR genes in the TCGA cohort. (C) Comparison of the prevalence of HRR mutations between the Chinese and TCGA cohorts. (D) The landscape of frequently mutated HRR genes in different molecular subtypes of muscle‐invasive bladder cancer.

Tumor samples from 343 Chinese UC patients from West China Hospital were analyzed, and a total of 221 HRR mutations were identified in 34.1% (117/343) of the patients. Among these mutations, 178/221 (80.5%) were missense mutations. The five most frequent HRR mutations were *BRCA2* (41/343, 12.0%), *ATM* (40/343, 11.7%), *BRCA1* (19/343, 5.5%), *CDK12* (16/343, 4.7%), and *RAD51C* (13/343, 3.8%) (Figure 1A). The mutation frequency of HRR gene was similar (32.2% vs. 35.1%) between UTUC and UCB. Similarly, no significant difference was observed in the mutation frequency of 15 specific HRR genes between these two groups (Figure S1A). For 822 UC patients from the TCGA cohort, we identified 461 HRR mutations in 31.4% of the patients, which was similar to our data. The most frequently mutated HRR genes in the TCGA cohort were *ATM* (96/822, 11.7%), *BRCA2* (61/822, 7.4%), *CDK12* (53/822, 6.4%), *BRCA1* (43/822, 5.2%), and *PALB2* (30/822, 3.6%) (Figure 1B). The prevalence of each HRR mutation was comparable between the two cohorts, except that *RAD51C* mutations were more frequently observed in the Chinese cohort (3.8% vs. 0.9%, FDR = 0.014, Figure 1C).

To further explore the HRR mutation patterns in different UC subtypes, we performed an integrative analysis using gene expression and mutation data from the TCGA cohort. Based on the consensus molecular classification, patients with muscle‐invasive bladder cancer (MIBC) were stratified into six molecular classes: basal/squamous (Ba/Sq, 35.8%), luminal papillary (LumP, 32.9%), luminal unstable (LumU, 13.2%), stroma‐rich (12.2%), luminal nonspecified (LumNS, 5.7%), and neuroendocrine‐like (NE‐like, 0.3%).^32^ Overall, LumU tumors were associated with numerically more HRR mutations, while LumNS and NE‐like tumors had relatively fewer HRR alterations (Figure S1B,C). For specific HRR mutations, *ATM* and *BRCA2* were still the most frequently mutated HRR genes across all subtypes (Figure S1D). Furthermore, *PALB2* mutation was relatively less frequent in Ba/Sq tumors (*p* = 0.056, Figure 1D).

### 3.2 Different mutational patterns between the HRR‐mutated and HRR‐wild type groups

**FIGURE 2:**
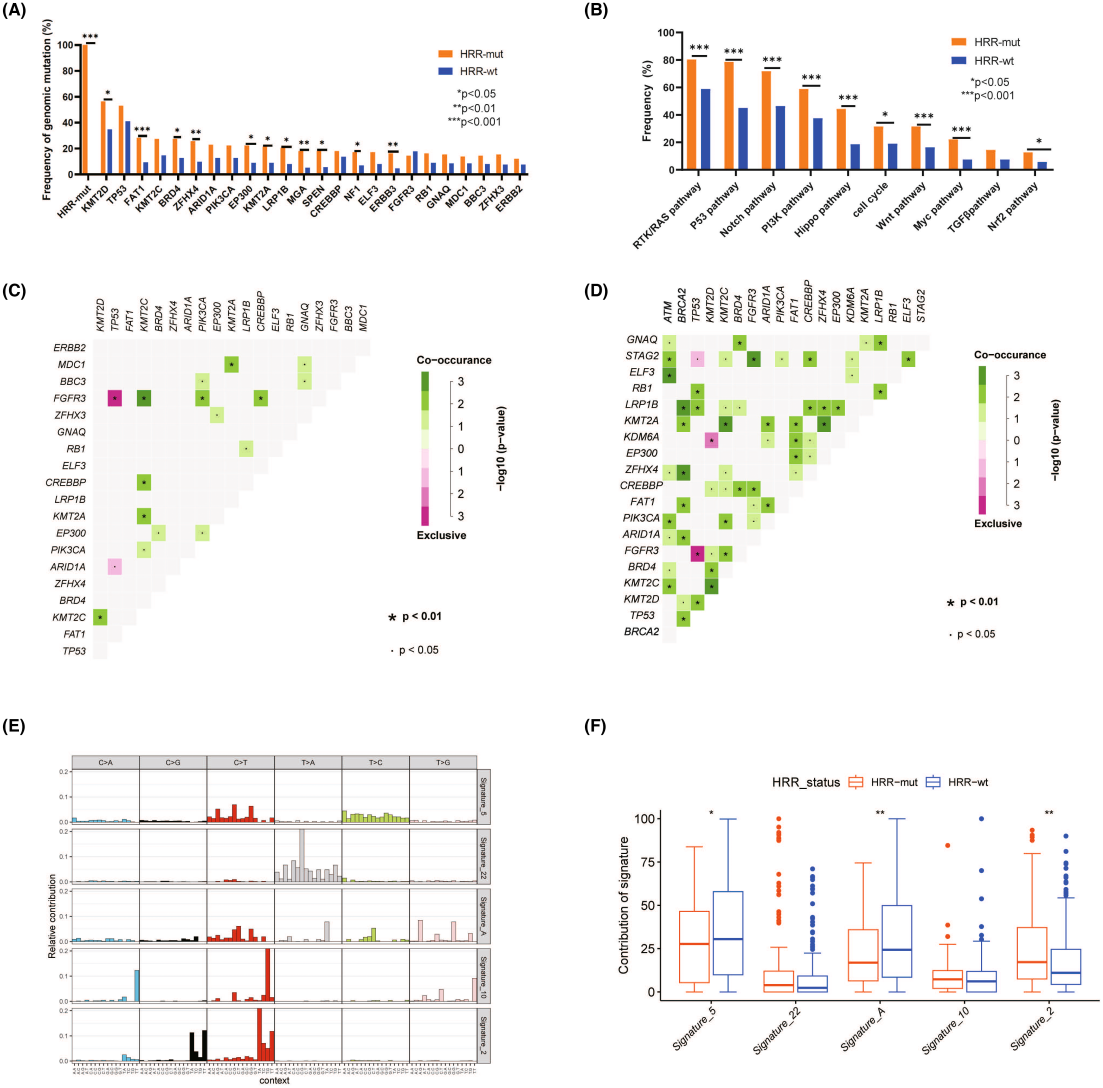
Mutational patterns of Chinese UC patients. (A) The frequency of the top 20 mutations in the HRR‐mut and HRR‐wt groups. (B) Comparison of oncogenic signaling pathways between the HRR‐mut and HRR‐wt groups. (C) Co‐occurring and exclusive mutations in the HRR‐wt group. (D) Co‐occurring and exclusive mutations in the HRR‐mut group. (E) Mutational signatures in the Chinese cohort. (F) Dominant mutational signatures between the HRR‐mut and HRR‐wt groups in the Chinese cohort.

To better understand how HRR mutations influence the genomic patterns in UC, we divided patients into HRR‐mutated (HRR‐mut) and HRR‐wild type (HRR‐wt) groups. In the Chinese HRR‐mut group, the most frequently mutated non‐HRR genes were *KMT2D* (56.4%), *TP53* (46.2%), and *FAT1* (35.0%). In the Chinese HRR‐wt group, the most commonly mutated genes were *TP53* (37.6%), *KMT2D* (35.4%), and *FGFR3* (18.6%) (Figure 2A). Among the 77 differentially mutated genes between the two groups, seven mutated non‐HRR genes were found exclusively in the HRR‐mut group, while there were no exclusive mutations in the HRR‐wt group (Table S3). Gene mutations associated with oncopathways such as RTK/RAS, P53, Notch, and PI3K pathway, showed higher frequency in the HRR‐mut group (Figure 2B). Similar results were observed in the TCGA cohort (Figure S2A,B).

We next performed co‐occurrence and mutual exclusion analyses for the top 20 genes in each group in the Chinese and TCGA cohorts. In the Chinese HRR‐mut group, co‐occurrence of mutations in *GNAQ/STAG2/ELF3/ZFHX4/PIK3CA/ARID1A/BRD4/KMT2C* with *ATM* mutations and co‐occurrence of mutations in *LRP1B/KMT2A/ZFHX4/FAT1/ARID1A/KMT2D/TP53* with *BRCA2* mutations were found (Figure 2D). Additionally, mutations in the *FGFR3* and *KMT2D* genes were significantly associated (co‐occurred) in both the Chinese (Figure 2C) and TCGA cohorts (Figure S2C). However, this co‐occurrence was not observed in the HRR‐wt group (Figure 2C and Figure S2D). Interestingly, *TP53* and *FGFR3* mutations were mutually exclusive both in the Chinese (Figure 2C,D) and TCGA cohorts (Figure S2C,D).

Subsequently, we carried out the mutational signature analysis based on NMF. In the Chinese cohort, signature 2 (APOBEC signature) was more likely to be present in HRR‐mut cohort compared to that in HRR‐wt cohort (*p* = 0.002, Figure 2E,F). Conversely, the HRR‐mut group had a lower proportion of signature 5 (ERCC2 mutation‐related signature) than the HRR‐wt group (*p* = 0.049, Figure 2E,F), indicating differential mechanisms might contribute to the development of UC based on the HRR mutation status. Analysis based on the TCGA data further validated our findings (Figure S2E,F). Interestingly, signature 22 (exposure to aristolochic acid), which accounted for 18.47% of the Chinese HRR‐mut cases, was not found in the TCGA cohort (Figure 2F, Figure S2F).

### 3.3 HRR mutations associated with a favorable response to immunotherapy in UC

**FIGURE 3:**
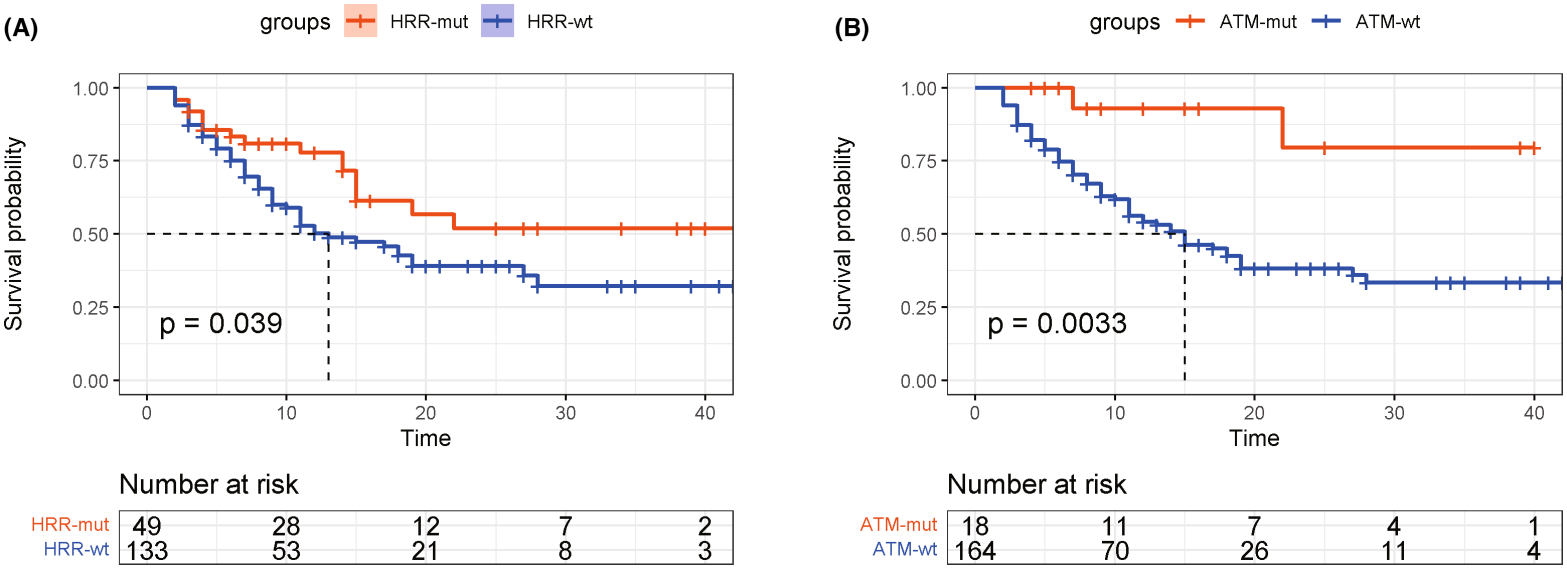
Survival outcomes of UC patients with and without HRR mutations after immunotherapy. (A) Overall survival of patients receiving immunotherapy stratified by HRR mutation status. (B) Overall survival of patients receiving immunotherapy stratified by ATM mutation status.

To evaluate the association between the presence of HRR mutations and the response to immunotherapy in UC, we analyzed the data of UC patients treated with immunotherapy from the MSK‐IMPACT cohort.^33^ We found that the presence of HRR mutations was significantly associated with improved prognosis in patients treated with immunotherapy (log‐rank *p* = 0.039; Figure 3A). For specific HRR genes, patients with *ATM* gene mutations gained significantly more survival benefits (log‐rank *p* = 0.003; Figure 3B). However, other HRR mutations showed no apparent correlations with the survival outcomes in patients with UC after immunotherapy (*p* > 0.05, Figure S3). Univariate Cox regression analysis further indicated that baseline characteristics such as age, gender, tumor location, and treatment type were not associated with prognosis in this setting, and only the overall HRR mutation status and *ATM* mutations were predictive of the efficacy of immunotherapy (Table S4).

### 3.4 HRR mutations associated with high tumor mutational burden and neoantigen burden

**FIGURE 4:**
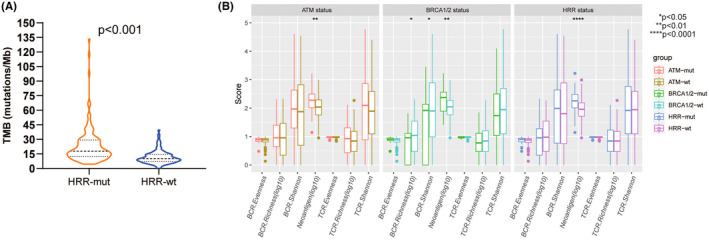
Relationship between HRR mutation status and tumor mutational burden (A) and immunogenicity (B).

Recently biomarker analyses have revealed a positive association between TMB and survival benefits in UC patients treated with avelumab.^34^ Thus, to understand the intrinsic mechanism of how HRR mutations influence immunotherapeutic responses, the association between HRR mutations and TMB was evaluated. We found that Chinese UC patients with HRR mutation harbored a higher TMB than those with HRR wildtype (median: 17.75 vs. 10.18, *p* < 0.001, Figure 4A). For specific HRR mutations, most of them were also associated with a higher TMB (*p* < 0.05, Figure S4). We further assessed the correlations between HRR mutations and immunogenicity based on the TCGA data. Unsurprisingly, patients with HRR mutations had a higher neoantigen burden than those without HRR mutations (*p* < 0.001, Figure 4B). This association was also observed in patients with *ATM* mutations and *BRCA1/2* mutations (Figure 4B). However, the antigen‐specific T‐cell receptor (TCR) and B‐cell receptor (BCR) repertoires were not associated with HRR mutation status (Figure 4B).

### 3.5 Association between HRR mutations and tumor immune microenvironment

**FIGURE 5:**
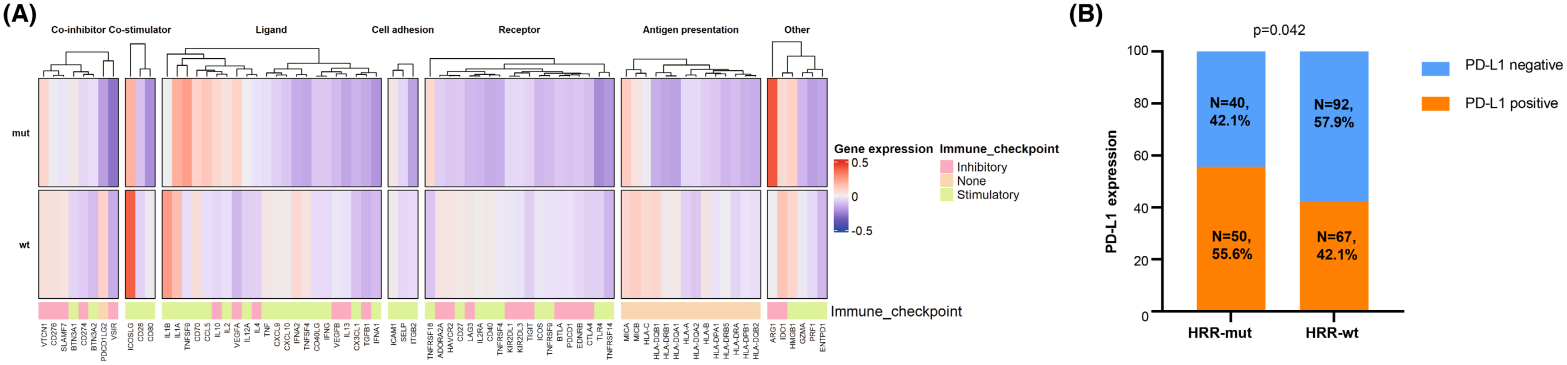
Association between immune‐related genes and HRR mutation status.

Additionally, we explored intratumoral immune states using immune expression signatures, including macrophages/monocytes, overall lymphocyte infiltration, the TGF‐β response, the IFN‐γ response, and wound healing.^35^ Unfortunately, there was no difference in these intratumoral immune signatures between the HRR‐mut and HRR‐wt groups (Figure S5). Next, the proportions of 22 immune cells were calculated by CIBERSORTx. However, no association between HRR gene mutation and the immune cell abundance was observed (Figure S6). Thus, we subsequently investigated whether HRR mutation status impacted the expression of immune‐regulatory genes. Seventy‐eight immune‐regulatory genes were explored and shown to be distinctly associated with HRR mutation status (Figure 5A). Patients with HRR mutations were enriched in a shared cluster with higher expression of ARG1, IL‐10, CCL5, TNFRSF18, and lower expression of IL1B and PDCD1LG2 (*p* < 0.05), which play a role in immune inhibition, the stimulation of ligands and receptors, and coinhibitory function. Given the crucial role of PD‐1/PD‐L1 axis in tumor immune regulation, we next explored the expression pattern of PD‐L1 using immunohistochemical staining. We found that tumors with HRR mutations had significantly higher expression of PD‐L1 than those without HRR mutations (55.6% vs. 42.1%, *p* = 0.042, Figure 5B).

## 4 DISCUSSION

HRR‐related genes and the HRD genomic scar assay are widely used to predict the response for poly‐polymerase (PARP) inhibitors in cancers.^36^ Additionally, an increasing number of reports have demonstrated that HRR genes might play a predictive role in multiple cancers treated with immunotherapy or platinum‐based chemotherapy.^37^ Nonetheless, the mutational patterns and immune signatures of the HRR genes and their predictive value for the immunotherapeutic response in UC remain to be clarified. To the best of our knowledge, this was the first study revealing the mutational landscape of UC with somatic HRR mutations, which manifested a distinct immune profile and was more sensitive to immunotherapy.

Our data showed that 34% of Chinese UC patients carried mutations in HRR genes, which was consistent with the findings from the TCGA cohort, indicating that the mutational pattern of HRR genes in UC was independent of race. Mutations in the *FGFR3* and *KMT2D* genes were significantly associated in both the Chinese and the TCGA cohorts. *KMT2D/KMT2C* are tumor suppressor genes, and prior studies reported that *KMT2D/KMT2C* deficiency could lead to DNA damage and genomic instability.^38^, ^39^ Moreover, distinct mutational signatures between the HRR‐mut and HRR‐wt groups were identified, which suggested different mechanisms of carcinogenesis. Signature 2, reflecting the activation of AID/APOBEC cytidine deaminases, was higher in the HRR‐mut group. Viral infection or tissue inflammation could cause the activation of AID/APOBEC cytidine deaminases in cancer, which may play a crucial role in DNA replication and positively modulate the immunological response.^40^ Signature 22, indicating exposure to aristolochic acids (AA), was observed only in the Chinese UC patients, which may be due to ethnic differences in drug usage.^41^ Lu et al. reported that patients harboring AA signature were related to AA exposure and showed a higher TMB, more neoantigens, and immune infiltration, implying the potential for immunotherapy.^41^ Based on these findings, we hypothesized that AA mutational signature might act as a novel marker of HRR and could predict the sensitivity to immunotherapy in Chinese UC patients. Recently, Lorenzo et al. also suggested the potential of long noncoding gene signatures to forecast the therapeutic response in bladder cancer, a concept that warrants further investigation.^42^

A prospective clinical trial of anti‐PD‐1/PD‐L1 monotherapy reported that DNA damage response and repair (DDR) gene alterations are independently associated with response to immunotherapy in mUC.^43^ Recently, an observational study enrolling 266 patients with non‐small cell lung cancer showed similar results.^44^ Moreover, a previous study analyzed several public cohorts and showed that patients with HRR mutations had better outcomes after anti‐CTLA‐4 treatment in advanced melanoma.^45^ However, the predictive value of HRR mutations for immunotherapeutic response in UC remains unknown. Our analysis revealed that patients with HRR mutations, especially those with *ATM* mutations, had improved overall survival than those without HRR mutations after immunotherapy. Interestingly, *BRCA*‐mut group failed to obtain more benefit from immunotherapy than *BRCA*‐wt group. A phase II, randomized, double‐blind, multicenter trial demonstrated that patients with *BRCA*‐mut ovarian cancer gained the most benefit from PARP inhibitors.^46^ Another recent trial suggested that PARP inhibitor was also an option for mUC patients harboring DDR gene mutations following chemotherapy, and a median PFS of 35.3 weeks with rucaparib was reported.^47^ Consistently, the BAYOU trial indicated that HRR gene alterations may serve as a biomarker for PARP inhibitor response in platinum‐ineligible patients with mUC.^48^ Thus, the HRR mutation status may serve as a biomarker not only for immunotherapy but also for PARP inhibitors in UC. More clinical evidence is needed to verify our findings and hypothesis.

The mechanism underlying the relationship between HRR mutations and the immunotherapeutic response is rather complicated and remains unclear. In our study, we showed that the HRR‐mut group was associated with a higher TMB and elevated neoantigen load than the HRR‐wt group in UC. It is striking that intratumoral immune states and immune cell abundance were irrelevant to the HRR status. Previously, whole‐exome sequencing revealed a robust association between higher TMB and clinical efficacy of PD‐1 blockade in advanced non‐small cell lung cancer (NSCLC) patients.^49^ A phase 3 trial further validated that ICB could extend PFS in NSCLC patients with high TMB.^50^ Recently, a study elucidated that high TMB was relevant to the improved OS in 1662 cancer patients treated with ICB. By contrast, the non‐ICB treated cohort was observed with no association between TMB and outcomes.^51^ Herein, we hypothesized that HRR alterations lead to a higher TMB, and more neoantigens are presented to major histocompatibility complex (MHC) molecules on T lymphocytes, facilitating the antitumor immune response. It is worth noting that immunogenic neoantigens are capable of escaping immune responses by chromosomal instability–induced copy number alterations, promoter hypermethylation, etc.^52^ Therefore, neoantigen‐based combination therapy may be a promising approach for UC patients with HRR alterations. A previous study reported a personalized neoepitope‐derived multipeptide vaccine complementary with immunotherapy (pembrolizumab), which induced very strong CD4+ as well as CD8+ T cell responses in patients with metastatic UC.^53^ A phase Ib trial in patients with high TMB metastatic tumors demonstrated that personalized neoantigen vaccine plus anti‐PD‐1 induced neoantigen‐specific CD4+ and CD8+ T cell responses, with antitumor capability.^54^

The present study is limited by its retrospective nature. In addition, the lack of transcriptome data in the Chinese cohort hinders the exploration of the immune landscape of Chinese UC patients. Last, due to limited clinicopathologic data from the TCGA and MSK‐IMPACT datasets, several known prognostic factors could not be explored. More in‐depth research with larger population size is needed to fully understand the molecular mechanisms and prognostic value of HRR mutations in UC.

## 5 CONCLUSIONS

In summary, we revealed the mutational landscape and immune profile of UC patients with somatic HRR mutations and demonstrated that HRR mutations were associated with a favorable response to immunotherapy, elevated TMB, increased neoantigen burden, and enriched PD‐L1 expression. These findings provide important insights into the potential clinical application of HRR mutations as a biomarker for immunotherapy in UC. However, further studies are warranted to validate and extend these findings, and additional mechanistic studies are needed to elucidate the underlying molecular mechanisms.

## AUTHOR CONTRIBUTIONS

**Junru Chen:** Formal analysis (lead); writing – original draft (lead); writing – review and editing (equal). **Yanfeng Tang:** Data curation (supporting); formal analysis (equal); writing – original draft (equal). **Huanhuan Liu:** Formal analysis (equal); methodology (equal); software (equal); writing – original draft (supporting). **Guangxi Sun:** Data curation (equal); investigation (supporting). **Haoyang Liu:** Data curation (equal). **Junjie Zhao:** Data curation (equal). **Zilin Wang:** Data curation (equal). **Yanrui Zhang:** Methodology (supporting); software (equal). **Feng Lou:** Methodology (supporting); software (equal). **Shanbo Cao:** Software (supporting). **Jiayue Qin:** Software (equal); supervision (supporting). **Huina Wang:** Software (equal); supervision (supporting). **Banghua Liao:** Conceptualization (equal); resources (lead); supervision (equal); writing – review and editing (supporting). **Hao Zeng:** Conceptualization (lead); funding acquisition (lead); supervision (equal); writing – review and editing (equal).

## FUNDING INFORMATION

This study was funded by National Natural Science Foundation of China (No. 82172785, 82103097, and 81974398), the Science and Technology Support Program of Sichuan Province (2021YFS0119), and the 1.3.5 Project for Disciplines of Excellence, West China Hospital, Sichuan University (No. 0040205301E21).

## CONFLICT OF INTEREST STATEMENT

All authors declare no financial or non‐financial competing interests.

## ETHICS STATEMENT

The Ethical Committee of West China Hospital approved this study. All participants provided written informed consent and consented to publish the article.

## Supporting information


Figures S1–S6

Tables S1–S4

## Data Availability

The data underlying this article will be shared on reasonable request to the corresponding author.
