# Supplementary material for: The mutational pattern of homologous recombination repair genes in urothelial carcinoma and its correlation with immunotherapeutic response
Source: Cancer Med. 2023 Nov 20;12(24):22370–80. doi: 10.1002/cam4.6725 (PMC10757100; doi:10.1002/cam4.6725)
Supplement: Supplementary file 1 — Figures S1–S6 [file CAM4-12-22370-s001.pdf]

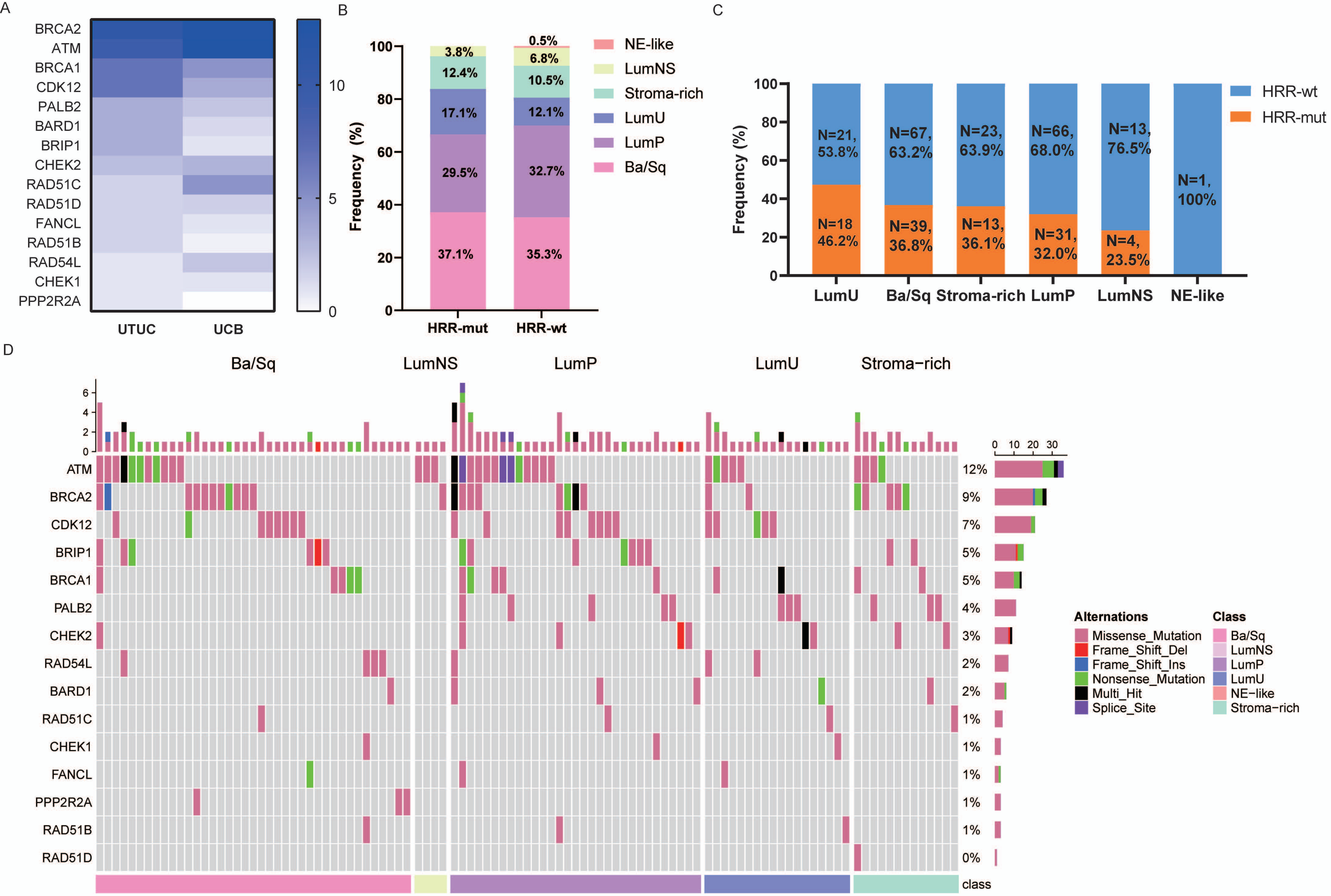


Figure S1. Mutational landscape of HRR genes in UC patients. (A) Comparison of HRR mutations between UTUC and UCB groups in the Chinese cohort. (B) The proportion of molecular subtypes according to HRR mutation status. (C) The proportion of HRR mutations in different molecular subtypes. (D) Landscape of frequently mutated HRR genes in different molecular subtypes.

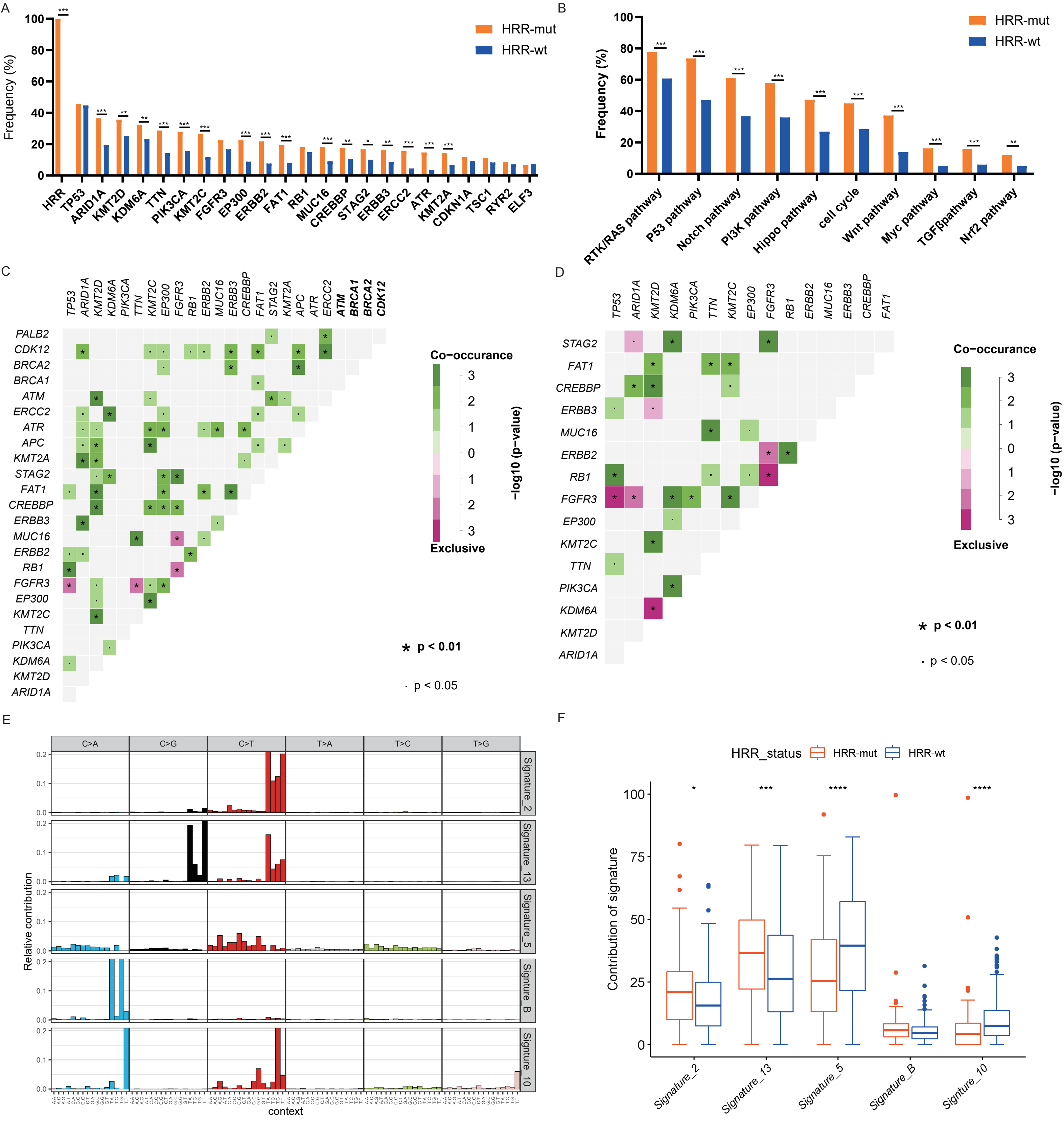


Figure S2. Mutational patterns of HRR genes in the TCGA cohort. (A) The frequency of the top 20 mutations in the HRR-mut and HRR-wt groups. (B) Comparison of oncogenic signaling pathways between the HRR-mut and HRR-wt groups. (C) Co-occurring and exclusive mutations in the HRR-mut group. (D) Co-occurring and exclusive mutations in the HRR-wt group. (E) Mutational signatures in the TCGA cohort. (F) Dominant mutational signatures between the HRR-mut and HRR-wt groups in the TCGA cohort.

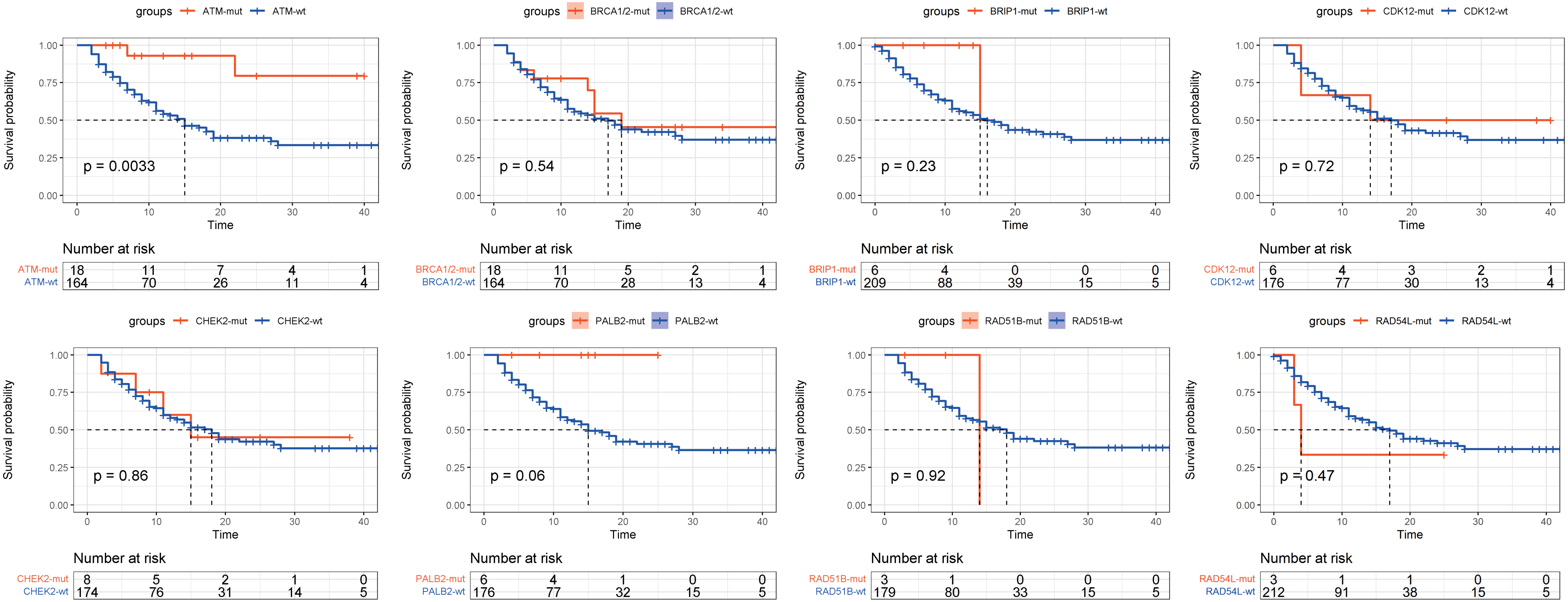


Figure S3. Survival outcomes of UC patients with and without specific HRR mutations after immunotherapy.

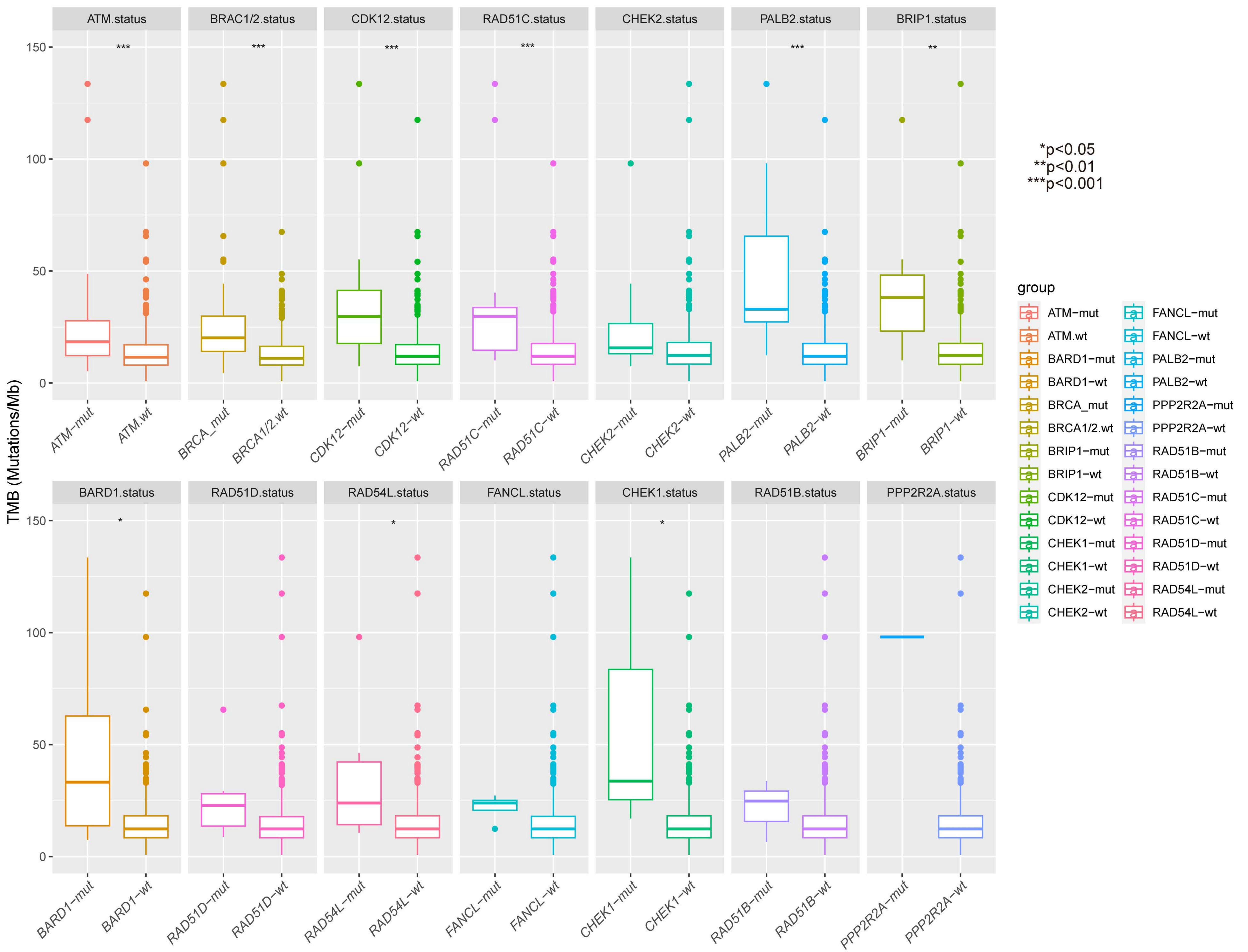


Figure S4. Relationship between specific HRR mutations and tumor mutational burden.

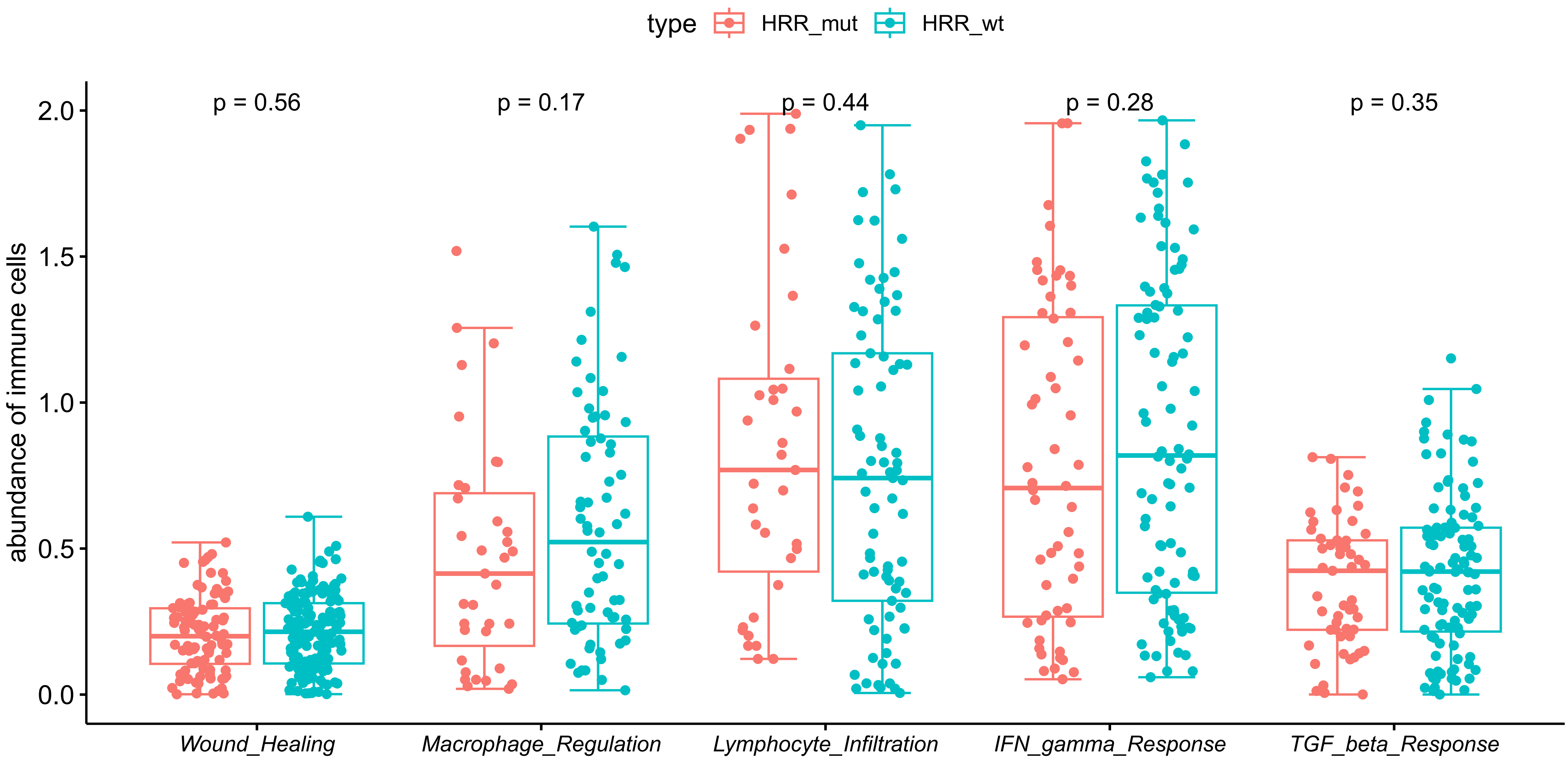


Figure S5. Comparison of expression levels of five intratumoral immune signatures between the HRR-mut and HRR-wt groups.

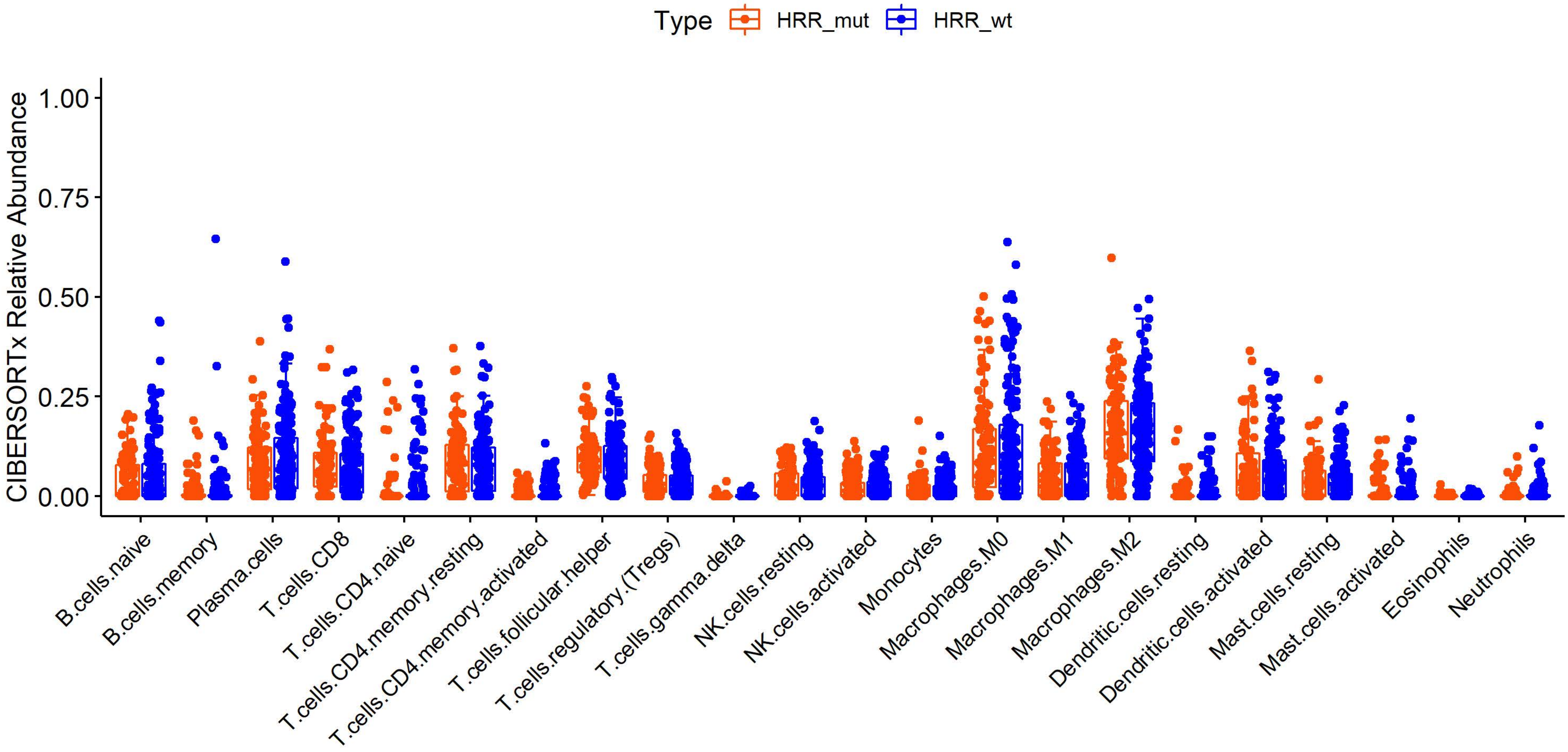


Figure S6. Association between immune cell aboudance and HRR mutation status by CIBERSORT method.
