# Supplementary material for: The mutational pattern of homologous recombination repair genes in urothelial carcinoma and its correlation with immunotherapeutic response
Source: Cancer Med. 2023 Nov 20;12(24):22370–80. doi: 10.1002/cam4.6725 (PMC10757100; doi:10.1002/cam4.6725)
Supplement: Supplementary file 2 — Tables S1–S4 [file CAM4-12-22370-s002.docx]

Table S1. Characteristics of UC patients.

| **Characteristics** | **Chinese cohort, n (%)** | **TCGA cohort, n (%)** |
| --- | --- | --- |
| **Total number** | 343 (100.0%) | 822 (100.0%) |
| **HRR status** |  |  |
| HRR mutation | 117 (34.1%) | 258 (31.4%) |
| HRR wildtype | 226 (65.9%) | 564 (68.6%) |
| **Tumor location** |  |  |
| Upper urinary tract | 118 (34.4%) | 116 (14.1%) |
| Lower urinary tract | 225 (65.6%) | 706 (85.9%) |
| **Sample type** |  |  |
| Fresh tissue | 164 (47.8%) | / |
| FFPE | 179 (52.2%) | / |
| **Tumor source** |  |  |
| Primary | 291 (84.8%) | 373 (45.4%) |
| Metastasis | 23 (6.7%) | 16 (1.9%) |
| Missing | 29 (8.5%) | 433 (52.7%) |
| **Metastatic status** |  |  |
| Metastatic disease | 89 (25.9%) | 98 (11.9%) |
| Localized disease | 254 (74.1%) | 143 (17.4%) |
| Missing | 0 (0.0%) | 638 (77.7%) |

HRR: Homologous recombination repair; FFPE: Formalin-fixed paraffin-embedding

Table S2. Characteristics of UTUC and UCB patients in the Chinese cohort

| **Characteristics** | **UTUC**  N = 118 | **UCB**  N = 225 |
| --- | --- | --- |
| **HRR status** |  |  |
| HRR mutation | 38 (32.20%) | 79 (35.11%) |
| HRR wildtype | 80 (67.80%) | 146 (64.89%) |
| **Gender** |  |  |
| Female | 42 (35.59%) | 41 (18.22%) |
| Male | 76 (64.41%) | 184 (81.78%) |
| **Median age** | 66 | 67 |
| **Sample type** |  |  |
| FFPE | 68 (57.63%) | 111 (49.33%) |
| Fresh tissue | 50 (42.37%) | 114 (50.67%) |
| **Tumor source** |  |  |
| Metastasis | 7 (6.93%) | 16 (7.51%) |
| Primary | 94 (93.07%) | 197 (92.49%) |
| Missing | 17 | 12 |
| Metastasis |  |  |
| No | 74 (76.29%) | 140 (67.96%) |
| Yes | 23 (23.71%) | 66 (32.04%) |
| Missing | 21 | 19 |

UTUC: upper tract urothelial carcinoma; UCB: urothelial carcinoma of the bladder;

HRR: Homologous recombination repair; FFPE: Formalin-fixed paraffin-embedding

Table S3. Significant difference in the frequency of genes mutation between HRR-mut and HRR-wt groups.

| gene | mut | wt | pvalue | p_fdr |
| --- | --- | --- | --- | --- |
| KMT2D | 66 | 82 | 0.000848639 | 0.010961582 |
| BRD4 | 32 | 30 | 0.002117137 | 0.016828526 |
| ZFHX4 | 30 | 23 | 0.000286773 | 0.005332067 |
| FAT1 | 33 | 22 | 1.81911E-05 | 0.000805604 |
| EP300 | 26 | 21 | 0.001470059 | 0.013020523 |
| KMT2A | 25 | 21 | 0.002769413 | 0.019078177 |
| LRP1B | 24 | 19 | 0.00197294 | 0.016095035 |
| NF1 | 20 | 16 | 0.005717301 | 0.030040057 |
| ERCC2 | 18 | 15 | 0.012261638 | 0.049365037 |
| SPEN | 21 | 13 | 0.000504404 | 0.008686964 |
| NCOR1 | 18 | 13 | 0.004356267 | 0.024115048 |
| MGA | 21 | 12 | 0.000253328 | 0.005332067 |
| ERBB3 | 19 | 11 | 0.000592669 | 0.009579152 |
| PTPRT | 18 | 11 | 0.001272153 | 0.012323978 |
| KDM5A | 15 | 10 | 0.0060194 | 0.031100232 |
| ANKRD11 | 18 | 9 | 0.000292404 | 0.005332067 |
| FBXW7 | 18 | 9 | 0.000292404 | 0.005332067 |
| NOTCH1 | 16 | 8 | 0.000677963 | 0.009579152 |
| PREX2 | 15 | 7 | 0.000679811 | 0.009579152 |
| PTPRD | 15 | 7 | 0.000679811 | 0.009579152 |
| SETD2 | 14 | 7 | 0.00155787 | 0.013052425 |
| PIK3C2G | 13 | 7 | 0.003486651 | 0.020038174 |
| RBM10 | 13 | 7 | 0.003486651 | 0.020038174 |
| ALK | 17 | 6 | 4.55679E-05 | 0.001410837 |
| GRIN2A | 13 | 6 | 0.00155076 | 0.013052425 |
| EML4 | 12 | 6 | 0.00355516 | 0.020038174 |
| EPHA5 | 12 | 6 | 0.00355516 | 0.020038174 |
| ARID5B | 12 | 6 | 0.00355516 | 0.020038174 |
| FAT3 | 11 | 6 | 0.007944671 | 0.03528054 |
| ATRX | 18 | 5 | 5.87752E-06 | 0.000303672 |
| KAT6A | 16 | 5 | 3.95951E-05 | 0.001410837 |
| BCORL1 | 11 | 5 | 0.003510774 | 0.020038174 |
| SOS1 | 10 | 5 | 0.008080382 | 0.03528054 |
| FANCA | 11 | 4 | 0.001359166 | 0.012392394 |
| LATS1 | 11 | 4 | 0.001359166 | 0.012392394 |
| FLT1 | 10 | 4 | 0.00803932 | 0.03528054 |
| IRS2 | 10 | 4 | 0.00803932 | 0.03528054 |
| PIK3C2B | 10 | 4 | 0.00803932 | 0.03528054 |
| ERBB4 | 11 | 3 | 0.001266447 | 0.012323978 |
| SLITRK1 | 10 | 3 | 0.003147386 | 0.020038174 |
| FOXO1 | 9 | 3 | 0.007662244 | 0.03528054 |
| EGFR | 9 | 3 | 0.007662244 | 0.03528054 |
| TERT | 9 | 3 | 0.007662244 | 0.03528054 |
| UPF1 | 9 | 3 | 0.007662244 | 0.03528054 |
| APOB | 10 | 2 | 0.001024661 | 0.011344463 |
| PDGFRA | 10 | 2 | 0.001024661 | 0.011344463 |
| MET | 10 | 2 | 0.001024661 | 0.011344463 |
| CSF1R | 10 | 2 | 0.001024661 | 0.011344463 |
| GLI1 | 9 | 2 | 0.002656105 | 0.018713466 |
| JAK2 | 9 | 2 | 0.002656105 | 0.018713466 |
| MAP3K1 | 9 | 2 | 0.002656105 | 0.018713466 |
| CD22 | 9 | 2 | 0.002656105 | 0.018713466 |
| RPS6KA4 | 9 | 2 | 0.002656105 | 0.018713466 |
| RICTOR | 8 | 2 | 0.006777787 | 0.033888935 |
| PIK3R2 | 8 | 2 | 0.006777787 | 0.033888935 |
| PDGFRB | 11 | 1 | 9.59026E-05 | 0.002477483 |
| DROSHA | 9 | 1 | 0.000721034 | 0.009718289 |
| BTK | 7 | 1 | 0.00527257 | 0.028180978 |
| RHBDF2 | 7 | 1 | 0.00527257 | 0.028180978 |
| BRCA2 | 41 | 0 | 8.82191E-21 | 2.73479E-18 |
| ATM | 40 | 0 | 2.99938E-20 | 4.64904E-18 |
| BRCA1 | 19 | 0 | 8.26864E-10 | 8.54426E-08 |
| CDK12 | 16 | 0 | 2.05048E-08 | 1.58912E-06 |
| RAD51C | 13 | 0 | 2.19828E-06 | 0.000136294 |
| CHEK2 | 10 | 0 | 5.0062E-05 | 0.001410837 |
| ETV5 | 10 | 0 | 5.0062E-05 | 0.001410837 |
| PALB2 | 9 | 0 | 0.0001413 | 0.003369464 |
| BRIP1 | 7 | 0 | 0.001125293 | 0.011628027 |
| STAT5B | 7 | 0 | 0.001125293 | 0.011628027 |
| BARD1 | 6 | 0 | 0.003185806 | 0.020038174 |
| RAD54L | 6 | 0 | 0.003185806 | 0.020038174 |
| RAD51D | 6 | 0 | 0.003185806 | 0.020038174 |
| NEGR1 | 5 | 0 | 0.009070955 | 0.036999948 |
| TLR4 | 5 | 0 | 0.009070955 | 0.036999948 |
| WT1 | 5 | 0 | 0.009070955 | 0.036999948 |
| KDM5C | 5 | 0 | 0.009070955 | 0.036999948 |
| ZNF804A | 5 | 0 | 0.009070955 | 0.036999948 |

Table S4. Univariate analysis of factors associated with overall survival after immunotherapy.

|  | **Univariate analysis** | | |
| --- | --- | --- | --- |
|  | **HR** | **95% CI** | **p value** |
| HRR mutation  Yes vs. No | 3.13 | 1.66 - 5.91 | 0.024 |
| ATM mutation  Yes vs. No | 14.02 | 1.94 - 101.27 | 0.009 |
| BRCA1/2 mutation  Yes vs. No | 1.58 | 0.72 - 3.46 | 0.544 |
| Age  (as continuous variable) | 0.99 | 0.97 - 1.02 | 0.777 |
| Gender  Male vs. Female | 1.53 | 0.80 - 2.93 | 0.197 |
| Tumor location  UTUC vs. UCB | 0.91 | 0.51 - 1.62 | 0.738 |
| Treatment type  ICI monotherapy vs. ICI combination therapy | 1.00 | 0.51 - 1.96 | 0.997 |

HR: hazard Ratio; CI: confidence interval; HRR: homologous recombination repair;

UTUC: upper tract urothelial carcinoma; UCB: urothelial carcinoma of the bladder;

ICI: immune checkpoint inhibitor
